# Crosstalk between tumor cells and lymphocytes modulates heparanase expression

**DOI:** 10.1186/s12967-019-1853-z

**Published:** 2019-03-29

**Authors:** Thérèse Rachell Theodoro, Leandro Luongo Matos, Renan Pelluzzi Cavalheiro, Giselle Zenker Justo, Helena Bonciani Nader, Maria Aparecida Silva Pinhal

**Affiliations:** 1 Biochemistry Department, Faculdade de Medicina do ABC, Av. Lauro Gomes, 2000, Santo André, SP 09060-870 Brazil; 2 Surgery Department (Head and Neck Discipline), Faculdade de Medicina da Universidade de São Paulo, Av. Dr. Arnaldo, 455, São Paulo, SP 01246-903 Brazil; 3 Biochemistry Department, Universidade Federal de São Paulo, Rua Três de Maio 100, São Paulo, SP 04044-020 Brazil; 4 Pharmaceutical Sciences Department, Universidade Federal de São Paulo, Rua Três de Maio, 100, São Paulo, SP 04044-020 Brazil

**Keywords:** Tumor microenvironment, Breast neoplasms, HPSE, HPSE2, Heparan sulfate, Proteoglycans

## Abstract

**Background:**

Heparanase (HPSE) is an endo-beta-glucuronidase that degrades heparan sulfate (HS) chains on proteoglycans. The oligosaccharides generated by HPSE promote angiogenesis, tumor growth and metastasis. Heparanase-2 (HPSE2), a close homolog of HPSE, does not exhibit catalytic activity. Previous studies have demonstrated that serum or plasma from breast cancer patients showed increased expression of both heparanases in circulating lymphocytes. The aim of this study was to better understand the mechanisms involved in the upregulation of heparanases in circulating lymphocytes.

**Methods:**

Lymphocytes collected from healthy women were incubated in the presence of MCF-7 breast cancer cells (co-culture) to stimulate HPSE and HPSE2 overexpression. The protein level of heparanases was evaluated by immunocytochemistry, while mRNA expression was determined by quantitative RT-PCR.

**Results:**

The medium obtained from co-culture of MCF-7 cells and circulating lymphocytes stimulated the expression of HPSE and HPSE2. Previous treatment of the co-culture medium with an anti-heparan sulfate proteoglycan antibody or heparitinase II inhibited the upregulation of heparanases in circulating lymphocytes. The addition of exogenous heparan sulfate (HS) enhanced the expression of both heparanases. Moreover, the co-cultured cells, as well as MCF-7 cells, secreted a higher number of exosomes expressing an increased level of HS compared to that of the exosomes secreted by circulating lymphocytes from women who were not affected by cancer.

**Conclusions:**

The results revealed that HS is likely responsible for mediating the expression of heparanases in circulating lymphocytes. HS secreted by tumor cells might be carried by exosome particles, confirming the key role of tumor cells, as well as secreted HS, in upregulating the expression of heparanases, suggesting a possible mechanism of crosstalk between tumor cells and circulating lymphocytes.

## Background

The extracellular matrix (ECM) acts as a physical scaffold for the microenvironment, facilitates interactions between different cell types and provides signals that elicit a variety of responses, such as cell proliferation, adhesion, migration, apoptosis, and angiogenesis [1]. Heparan sulfate (HS) is involved in several biological functions due to its interactions with different proteins in the ECM. The glycosaminoglycan chains of heparan sulfate proteoglycan (HSPG) can be degraded by heparanase (HPSE), generating oligosaccharides that intensify the effects of growth factors, cytokines, and angiogenic factors, triggering cell proliferation, cell differentiation, and angiogenesis, which favor tumor development [2–5]. Unlike HPSE, heparanase-2 (HPSE2) has no catalytic activity but binds to heparan sulfate with high affinity and appears to modulate HPSE enzymatic activity [6]. HPSE stimulates the synthesis and shedding of syndecan-1 proteoglycan (Syn-1) [7, 8]. Secreted Syn-1 binds to tumor-derived growth factors in the tumor microenvironment and promotes tumor progression [8]. The combination of HPSE enzymatic activity and Syn-1 shedding provides a powerful tool to better understand tumor development and metastasis [9, 10]. The tumor metastatic events depend on the cumulative ability of cancer cells to create an appropriate, distinct microenvironment in the primary tumor site and systemic circulation [11]. The primary tumor microenvironment is composed of different stromal cell types in addition to neoplastic and hematopoietic cells, such as bone marrow-derived cells (BMDCs), including macrophages, mast cells, mesenchymal stem cells (MSCs), myeloid cell-derived suppressor cells (MDSCs) and lymphocytes [12]. Triple-negative breast cancer represents the worst type of breast cancer and can be classified into four different subtypes: basal-like (BL), mesenchymal (M), luminal androgen receptor (LAR), and immunomodulatory (IM). Some triple-negative breast cancers have high levels of immune cell infiltration, and other triple-negative tumors have low levels of immune cell infiltration. In a recent study, it was demonstrated that among all triple-negative breast cancers, rich lymphocyte infiltration represented a good prognosis that could derive benefit from immune checkpoint inhibitor therapy, enhancing the anticancer activity of the immune system [13]. Tumor-infiltrating lymphocytes differ in triple-negative breast cancer. Furthermore, the association between the IM subtype and lymphocyte infiltration may influence the response to chemotherapy [14]. Infiltrating tumor lymphocytes improve immunological activity against tumors and can also influence prognosis. A previous study demonstrated a significant role for CD4^+^ T cells as enhancers of lung metastases from breast carcinomas by preventing the reduction or destruction of malignant cells due to the cytotoxic mechanisms present in CD4^+^ T cells [15]. Thus, increasing T cell traffic to the tumor may be a good strategy to enhance responses to immunotherapy in cancer treatment [16]. The expression of HPSE in the microenvironment can modulate the transmigration and extravasation of hematopoietic stem cells and bone marrow progenitor cells, thus affecting the hematopoietic system [17]. Moreover, Theodoro and colleagues observed increased levels of HPSE and HPSE2 in peripheral blood mononuclear cells (PBMCs) of breast cancer patients [18]. It is also conceivable that the exosomes secreted by tumor cells can modulate tumor metastasis and may be involved in microenvironmental signals. During the initial stages, exosomes remain at the primary site of the tumor, and they are secreted into the peripheral blood with tumor progression, subsequently acting on different tissues where tumor metastases develop [19, 20]. Exosome secretion often increases during tumor cell trafficking, generating a more aggressive phenotype. It has been observed that tumor exosomes can promote a significant acceleration of the metastatic process and increase the recruitment of MDSCs [21]. Higher exosome levels have been observed in cancer patients’ body fluids compared to those of healthy controls [22]. The highly malignant nature of tumors and the concentrations of extracellular vesicles in the circulation of patients affected by cancer suggest a prominent role of extracellular vesicles in promoting tumor progression and evading immune surveillance. In recent work, high and low extracellular vesicle concentrations have been shown to have differential effects that dictate the modulatory effects on PBMCs [23].

Exosomes contain miRNA, mRNA, proteins, and lipids, thereby providing a method for intercellular communication [24]. Recently, it has been shown that heparan sulfate from Syn-1 is an active modulator of its own shedding by epithelial and tumor cells [25]. Through interactions with syntenin (syn1-cytoskeleton binding protein), syndecan proteoglycans influence the biogenesis of exosomes [26]. There is also a direct relationship between increases in the secretion of exosomes and HPSE enzymatic activity. Furthermore, it was demonstrated that the protein composition of exosomes was modified by an increase in heparanase in an aggressive type of tumor. Exosomes secreted by tumor cells, together with high levels of heparanase, not only alter the behavior of tumor cells but also promote alterations to nonneoplastic host cells [27]. We were interested in investigating the mechanism of interaction between tumor cells and lymphocytes that activates heparanase expression in malignant neoplasms. Belting and colleagues highlighted a role for HSPGs in exosome uptake. Indeed, HSPGs appear to function as internalizing receptors for cancer cell-derived exosomes and to be required for their functional activity, namely, in driving cancer cell migration [28]. Thompson et al. reported that heparanase stimulated exosome production and affected the composition of exosomes [27]. Roucourt et al. provided evidence that heparanase activated the syndecan-syntenin-ALIX pathway, which supported the biogenesis of exosomes, affecting specific exosomal cargo [29, 30]. Since heparanase is known to be involved in tumor progression, several inhibitors of this enzyme have been produced as novel cancer therapeutics. Among the heparanase-inhibiting compounds, we highlight PI88 (a highly sulfonated mannan oligosaccharide), PG545 (a synthetic mixture of oligosaccharides derived from heparin) and SST0001 (a modified heparin saccharide with 100% N-acetylation and 25% glycol split that is also known as roneparstat), which are currently in clinical trials [31]. An improved understanding of the molecular contexts favoring the action of these agents against cancer would allow the full application of their potential.

The results revealed that heparan sulfate is likely responsible for mediating HPSE and HPSE2 expression in circulating lymphocytes. In addition, exosomes may play a role in heparanase upregulation by carrying secreted heparan sulfate from tumor cells, thus suggesting a mechanism of crosstalk between tumor cells and circulating lymphocytes.

## Methods

### Samples

Peripheral blood lymphocytes were obtained from women with breast cancer and women who were not affected by cancer (healthy women). Peripheral blood samples (5 mL) were collected using ethylenediaminetetraacetic acid (EDTA). Peripheral blood mononuclear cells (PBMCs) were obtained after centrifugation (3000×*g* for 30 min) in the presence of Ficoll Histopaque (Ficoll Hypaque; Organon Teknika^®^, Durham, NC, USA). PBMCs were counted in a Neubauer chamber and adjusted to a final concentration of 1 × 10^6^ cells/mL for all assays.

### Cell culture

The breast cancer cell line (MCF-7 cells) or lymphocytes collected from breast cancer patients or healthy women were maintained at 5% CO_2_ atmosphere and 37 °C in DMEM (Dulbecco’s Modified Eagle Medium) (Life Technologies^®^, Carlsbad, California, USA), containing 10% fetal bovine serum (FBS) (Invitrogen by Life Technologies^®^, Carlsbad, California, USA), 50 U/mL penicillin G (Invitrogen) and 50 mg/mL streptomycin sulfate (Invitrogen). For each assay, lymphocytes and plasma samples were obtained from different healthy donors or cancer patients.

### Flow cytometry

The cells analyzed by flow cytometry (FACSCalibur^®^, BD Biosciences, New Jersey, USA) were previously permeabilized with 0.01% saponin in 0.1 M sodium phosphate buffer for 15 min, followed by specific antibody labeling. To determine the percentage of T-lymphocytes, B-lymphocytes and NK (natural killer) cells in the PBMC fraction, the following antibodies were used: anti-CD3 (human anti-mouse FITC clone HIT3a), anti-CD4 (PE mouse anti-human clone RPA-T4), anti-CD19 (PE mouse anti-human clone 4G7) and anti-CD56 (PE CyTM mouse anti-human clone B159). All antibodies were obtained from BD Bioscience Pharmingen^®^, Inc. (California, USA) and used at a final dilution of 1:500. To analyze the heparanase isoform samples, anti-HPA1 C-20 and anti-HPA2 C-17 were used (Santa Cruz Biotechnology Inc., California, USA) for HPSE and HPSE2, respectively.

### Co-culture assay

The lymphocytes (1 × 10^6^ cells) were co-cultured for 18 h with 1 × 10^6^ MCF-7 cells maintained in DMEM, 5% CO_2_ and 37 °C. The co-culture medium was collected for other assays.

### Lymphocyte activation in vitro

Lymphocytes were incubated with conditioned medium from MCF-7 cells, MCF-7 cells (co-culture), plasma collected from healthy women or plasma obtained from breast cancer patients for 4 h at 37 °C with constant stirring (100 rpm). Lymphocyte activation assays were also performed in the presence of anti-syndecan-1 (clone CD138 BB4 MCA681) diluted 1:50 (AbD Serotec^®^, Bio-Rad Company Co., Oxford, UK), or the co-culture medium was previously treated with heparitinase II (HTase II from *Flavobacterium heparinum*). The digestion with HTase II was performed in 20 mM Tris–HCl buffer containing 50 mM NaCl and 4 mM CaCl_2_, pH 7.5. The activation of lymphocytes was also performed in the presence of bovine lung heparan sulfate (50 µg/mL) prepared in the Molecular Biology Laboratory at Universidade Federal de São Paulo as described [32].

### Immunocytochemistry

Heparanase immunostaining was performed using anti-HPA1 C-20 and anti-HPA2 C-17 antibodies (Santa Cruz Biotechnology^®^, Santa Cruz, CA, USA). A biotin-avidin-peroxidase complex was used to develop the reaction with 3,3′-diaminobenzidine used as the chromogen. Two independent observers scored 300 cells/slide as positive or negative according to the presence of staining for each of the above mentioned antibodies. The immunocytochemistry staining was analyzed by digital quantification. The slides were examined under a light microscope (Nikon Eclipse^®^ TS100) to identify the areas that best represented typical immunostaining (*hot spots*). In each case, the quantitation was performed by digital analysis, and the values are expressed as the expression index (EI), following the methodology described by Matos and colleagues [33]. Photomicrographs of 640 × 480 pixels were obtained from consecutive nonoverlapping fields at 400× magnification with a digital camera (Nikon Coolpix^®^ 4300) using the same parameters. The images were analyzed by a processing system and image analysis was performed using ImageLab (Softium Informática^®^, São Paulo, Brazil) using a micrometer scale (µm).

### Profile of sulfated glycosaminoglycans (GAGs)

Lymphocytes in culture were labeled using 100 µCi/mL of sodium sulfate [Na^35^SO_4_] for 18 h at 37 °C and 5% CO_2_. After metabolic labeling, the medium was collected, and lymphocytes were lysed using 3.5 M urea. The cellular fraction and culture medium were subjected to proteolysis with protease P126, also known as maxatase, at 4 mg/mL (Biocon do Brasil Indústria Ltda, RJ, Brazil) for 24 h. An aliquot of the cell fraction that did not undergo proteolysis was used to quantify the total protein using 117 µM Coomassie™ Brilliant Blue G (Sigma-Aldrich^®^, Co. St. Louis, MO, USA). The identification of GAGs was performed by gel electrophoresis using 0.55% agarose with acetate buffer and 0.05 M 1,3-diaminopropane (PDA), pH 9.0, at 100 volts for 1 h at 4 °C. The gel was dried, stained and subjected to autoradiography by exposure to X-ray film. The film was imaged using the scanning apparatus Cyclone^®^ (Packard Instrument Company, Meriden, CT, USA). For quantification of ^35^S-GAGs, the radioactive bands were excised from the agarose gels and were counted in 5 ml of Ultima Gold (PerkinElmer) using a liquid scintillation spectrometer.

### Enzymatic degradation of sulfated glycosaminoglycans

Approximately 10,000 cpm of [^35^S]-glycosaminoglycans, synthesized by lymphocytes, was incubated with 0.1 U of the specific enzymes chondroitinase AC from *Flavobacterium heparinum*, chondroitinase ABC from *Proteus vulgaris* [34] and heparitinase II from *Flavobacterium heparinum* [35].

### Quantitative RT-PCR (qRT-PCR)

Total RNA extraction was obtained using the TRIzol^®^ reagent (Life Technologies™ by Ambion, CA, USA), following the manufacturer’s instructions. Reverse transcription was performed using the reverse transcriptase enzyme ImPromII^®^ (Promega Co.^®^, WI, USA) according to the manufacturer’s instructions to obtain complementary DNA (cDNA). The mRNA expression of heparanase isoforms (HPSE and HPSE2) and Syn-1 were analyzed using the following primers: HPSE forward, 5′TGGCAAGAAGGTCTGGTTAGGAGA3′ and reverse, 5′GCAAAGGTGTCGGATAGCAAGGG3′; HPSE2 forward, 5′AGACAGAG CTGCAGGTTTGAAGGA3′ and reverse, 5′AGCTTAGGAAATCGAGCCAGCCAT3′; Syn-1 forward, 5′AGGGCTCCTGCACTTACTTGCTTA3′ and reverse, 5′ATGTGCA GTCATACACTCCAGGCA3′. The expressions of the endogenous genes 60S ribosomal protein L13A (RPL13a) and glyceraldehyde-3-phosphate dehydrogenase (GAPDH) were analyzed using the following primers: RPL13a forward, 5′TTGAGGACCTCTGTGTATTTGTCAA3′ and RPL13a reverse, 5′CCTGGAGGAGAAGAGGAAAGAGA3′; GAPDH forward, 5′TCGACAGTCAGCCGCATCTTCTTT3′ and GAPDH reverse, 5′GCCCAA TACGACCAAATCCGTTGA3′. The values are expressed as −ΔCt. Amplification was performed using Maxima^®^ reagent SYBR^®^ Green qPCR Master Mix (2x) (Applied Biosystems^®^, CA, USA) using a 7500 Real Time PCR Cycler^®^ (Applied Biosystems^®^, CA, USA).

### Exosome Analysis

Total exosome purification was performed from overnight conditioned culture medium collected from MCF-7 cells, non-activated lymphocytes (obtained from women who were not affected by cancer) and activated lymphocytes (obtained by previous co-culture with MCF-7 cells). The supernatant containing the cell-free media was used to obtain exosomes using a Total Exosome Isolation kit (Invitrogen^®^ by Life Technologies^®^), following the manufacturer’s protocol. Exosome preparation samples underwent total RNA extraction with TRIzol^®^ (Life Technologies^®^, CA, USA). Reverse transcription and quantitative RT-PCR were performed as previously described.

### Confocal Microscopy

A sample of 100 µL of each exosome preparation was submitted to cytocentrifugation at 250×*g* for 2 min. The exosome samples were fixed with methanol for 5 min on glass slides. The exosomes were permeabilized with 0.01% saponin and incubated with primary antibodies in the presence of 1% BSA (bovine serum albumin) for 2 h at room temperature. The slides were incubated overnight at 4 °C with the primary antibody anti-CD63 GTX44174 conjugated with FITC (CLBGran/12 with FITC GeneTex^®^) at a 1:20 dilution. For the detection of HS, the exosomes were incubated with an anti-HS primary antibody (MAB204; Millipore, Massachusetts, USA) diluted with PBS (1:250) for 2 h at room temperature. The exosomes were then incubated with Alexa Fluor^®^ 647 (1:250) in PBS for 1 h at room temperature. Coverslips were mounted on glass microscope slides using Fluoromount-G. The images were captured with a confocal laser scanning microscope (Leica TCS SP8) equipped with a Plan-Apochromat 63x objective (numerical aperture 1.4) under oil immersion. The pinhole device was adjusted to capture the fluorescence of one Airy unit. The images represented the maximum intensity projections that corresponded to the z-series of confocal stacks. The fluorescence of exosomes (anti-HS-Alexa Fluor^®^ 647 and anti-CD63-FITC) was adjusted to the mean threshold, and exosomes were counted using a particle analysis plugin in the ImageJ software (NIH-ImageJ, U. S. National Institutes of Health, Bethesda, Maryland, USA). The exosomes were counted in five different fields, and the assay was carried out in triplicate.

### Statistical analysis

The quantitative variables are described as the median and range (difference between maximum and minimum values). Absolute and relative frequencies are used for the qualitative variables. The distributions were defined as nonparametric by the Shapiro–Wilk test. The Mann–Whitney test was used for comparisons between two groups; for comparisons among three or more groups, the Kruskal–Wallis test, with the Dunn auxiliary test in comparisons of subgroups, was used. For all analyses, a statistical significance level of 5% was adopted (P ≤ 0.05), and Prism^®^ software version 5.0 (GraphPad Prism Software Inc.^®^, California, USA) was used.

## Results

As described previously, PBMCs were allowed to adhere in cell culture in the presence of phorbol ester (PMA), and after 24 h, non-adherent cells were incubated with the plasma of breast cancer patients; the expression of HPSE and HPSE2 was significantly enhanced in these cells. Since PMA promotes monocyte differentiation in macrophages, these results suggested that monocytes were not responsible for the overexpression of the heparanase isoforms (data not shown).

To investigate which cells were present in the PBMC population, the PBMC fraction was analyzed by flow cytometry using different antibodies. As shown in Table 1, approximately 70% of PBMCs were classified as T-lymphocytes, 7% as B-lymphocytes and 7% as natural killer cells (NK cells). It is interesting to note that independently of the incubation with the different types of conditioned medium, there were no changes in the PBMC population (Table 1). Based on these results, the PBMC population was classified as T-lymphocytes.

**Table 1:** Characterization of the peripheral blood mononuclear cells (PBMC)

	T-lymphocytes (%)		B-lymphocytes (%)		NK cells (%)	
	Median	Range	Median	Range	Median	Range
Peripheral blood mononuclear cells (PBMC)	71.2	6.2	7.2	2.4	8.3	1.7
PBMC + MCF-7 culture medium	68.4	12.7	5.5	2.2	5.3	0.9
PBMC + co-culture medium	71.6	8.1	8.1	1.5	5.3	3.6

Co-culture = lymphocytes from a healthy woman in the presence of MCF-7 cells for 18 h

PBMC was incubated with culture medium of MCF-7 cells and co-culture for 4 h at 37 °C and 5% CO_2_

Notably, MCF-7 cells did not behave differently during co-culture with lymphocytes for 18 h. No differences were observed in the profile of glycosaminoglycans, proliferation and viability of MCF-7 cells before and after co-culture assays (data not shown).

**Fig. 1:**
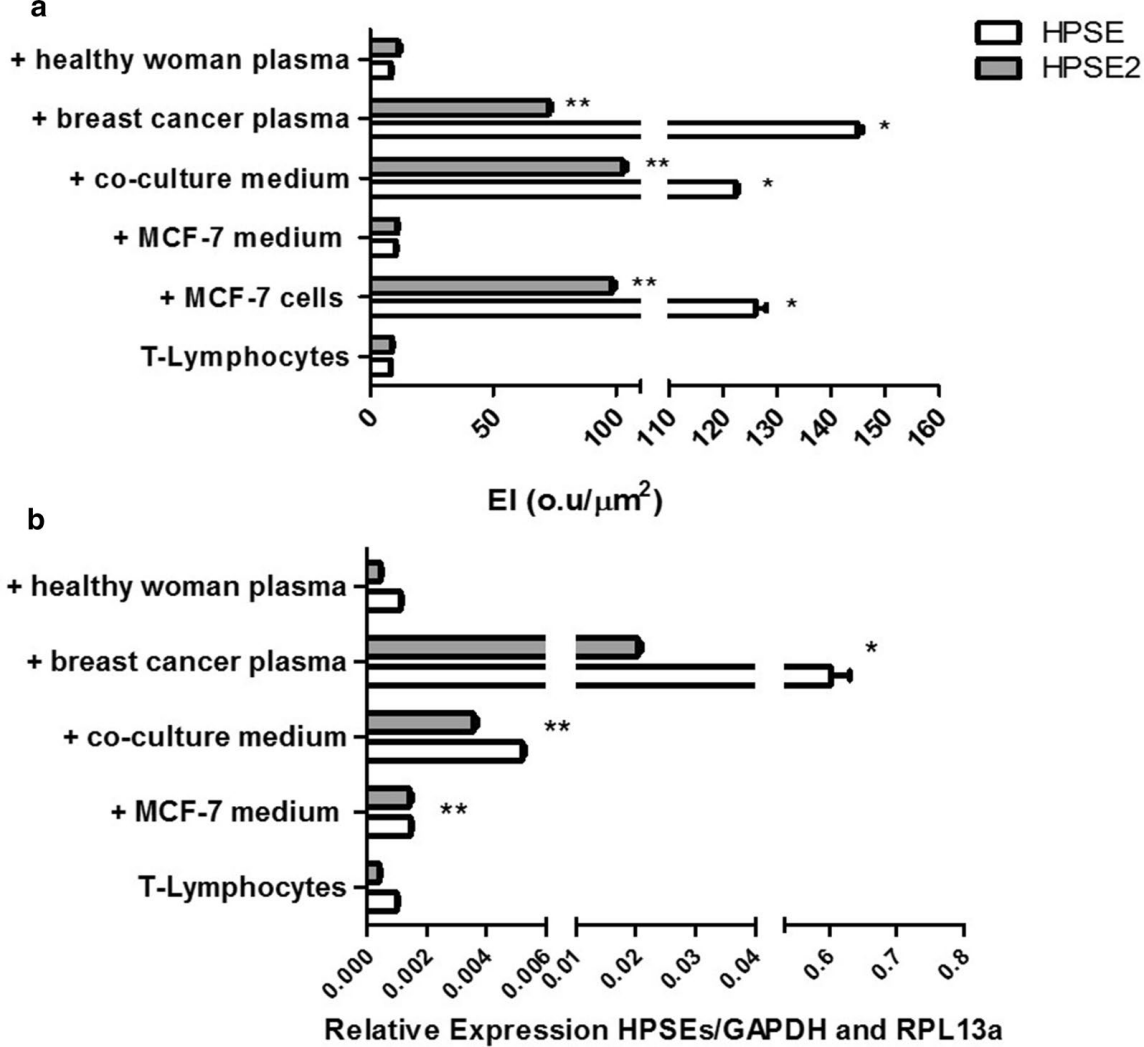
Expression of heparanase isoforms in lymphocytes. The lymphocytes were incubated as described for 4 h, at 37 °C. **a** Expression index (EI) obtained by digital quantification of immunohistochemistry reaction indicating protein level of both heparanases (HPSE and HPSE2). **b** The values represent mRNA expression of both heparanases, HPSE and HPSE2, using quantitative RT-PCR (qRT-PCR). The Ribosomal Protein L13a (RPL13a) and glyceraldehyde-3-phosphate dehydrogenase (GAPDH) were reference endogenous genes. The assays were performed with the peripheral blood mononuclear cell fraction obtained from healthy women. T-lymphocytes, lymphocytes obtained from a woman non-affected by cancer. The circulating lymphocytes were incubated for 4 h at 37 °C as indicated, + MCF-7 cells, with MCF-7 cells; + MCF-7 medium, with conditioned medium obtained from MCF-7 cells; + co-culture medium, with conditioned medium from co-culture; + breast cancer plasma, with plasma obtained from breast cancer patient; + healthy woman plasma, with plasma from healthy women. It is important to note that each value represents the median and range of triplicate assays that were performed using lymphocytes obtained from one healthy donor and plasma samples that were collected from three healthy donors, as well as three different patients with breast cancer. RT-PCR reaction was performed in triplicate for each independent sample. *p < 0.0001 and **p < 0.05 by the Kruskal–Wallis test with Dunn auxiliary test. *HPSE and **HPSE2 expression compared to T-lymphocytes without activation assay

The immunocytochemistry analysis clearly demonstrated the increased expression of both isoforms of heparanase (HPSE and HPSE2) in T-lymphocytes exposed to the plasma of breast cancer patients or to MCF-7 cells (Fig. 1a). Furthermore, the increased intensity of HPSE and HPSE2 protein expression was also observed when T-lymphocytes were previously incubated with the conditioned medium from co-culture (medium collected from co-culture of T-lymphocytes from healthy woman and MCF-7 cells). In contrast, the medium collected from MCF-7 cell culture, as well as the plasma of healthy women, was not able to induce such upregulation (Fig. 1a).

There was a significant increase in the mRNA level of both heparanases (HPSE and HPSE2) when T-lymphocytes were exposed to plasma of breast cancer patients or to co-culture medium, confirming the previous results obtained by immunocytochemistry (Fig. 1b).

**Fig. 2:**
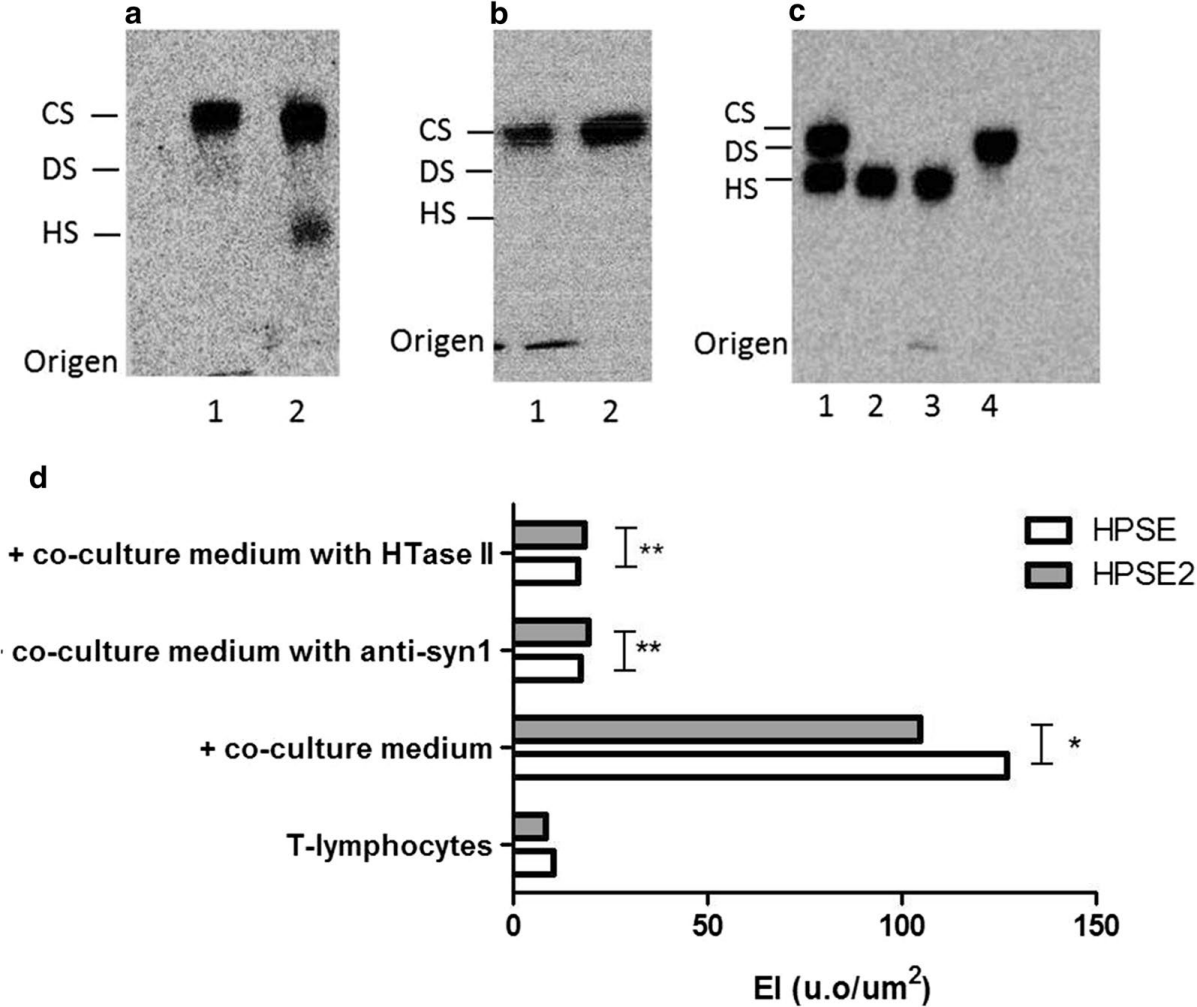
T-lymphocytes modulation by heparan sulfate (HS) and syndecan-1 (syn-1). Profile of sulfated glycosaminoglycans (GAG) synthesized by circulating lymphocytes. Circulating lymphocytes were maintained in culture for 4 h at 37 °C and GAG were identified and quantified by agarose gel electrophoresis after [^35^S]-sulfate labeling. **a** T-lymphocytes from a breast cancer patient; **b** T-lymphocytes of a woman not affected by cancer (healthy woman); **c** co-culture of T-lymphocytes from a healthy woman and MCF-7 cells; 1, cellular fraction; 2, medium; 3, digestion using chondroitinase AC; 4, digestion with heparitinase II. **d** The values represent digital quantification of immunocytochemistry assays performed in triplicates using lymphocytes obtained from three different healthy donors. T-Lymphocytes, circulating lymphocytes collected from a woman not affected by neoplasia; Lymphocytes were previously incubated for 4 h at 37 °C as indicated; + co-culture medium, in the presence of co-culture medium; + co-culture medium with anti-syn1, with co-culture medium in the presence of anti-syndecan-1 antibody (MCA681-CD138); + co-culture medium with HTase II, with co-culture medium in the presence of the enzyme heparitinase II. *p < 0.0001 and **p < 0.05 Kruskal–Wallis test with Dunn auxiliary test

We decided to investigate the profile of glycosaminoglycans (GAGs) synthesized by T-lymphocytes from breast cancer patients compared to those from control T-lymphocytes (lymphocytes obtained from healthy women). As shown in Fig. 2a, chondroitin sulfate was the major sulfated glycosaminoglycan present in the cellular fraction of breast cancer patients’ T-lymphocytes, while chondroitin sulfate and heparan sulfate were secreted into the medium. Moreover, control T-lymphocytes collected from a woman who was not affected by cancer presented only chondroitin sulfate in the cellular fraction as well as secreted into the medium (Fig. 2b). Digestion with chondroitinase AC, chondroitinase ABC, and heparitinase II confirmed the presence of chondroitin sulfate and heparan sulfate (Fig. 2c). Anti-syndecan-1 and heparitinase II abolished the stimulatory effect on HPSE and HPSE2 expression in circulating T-lymphocytes, as shown in Fig. 2d. Therefore, in contrast to lymphocytes from healthy individuals, lymphocytes from breast cancer patients, as well as lymphocytes activated by tumor cells, secreted heparan sulfate.

The use of the 3G10 antibody demonstrated the presence of syndecan-1 in the co-culture medium, whereas this proteoglycan was absent from the conditioned medium of MCF-7 cells and from the plasma from a woman who was not affected by cancer (data not shown).

**Fig. 3:**
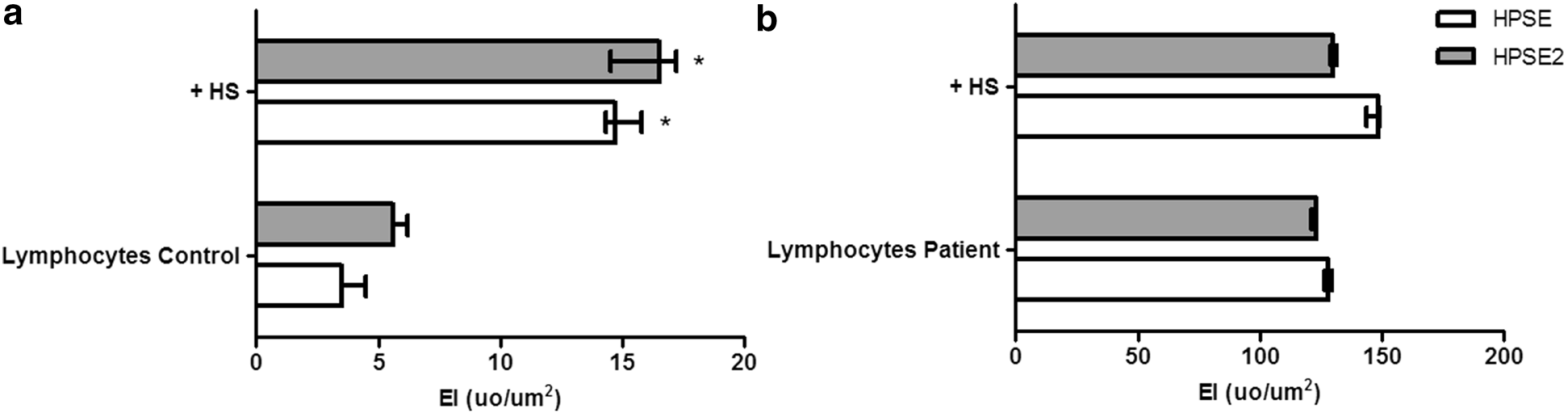
Effect of exogenous heparan sulfate on the expression of both heparanases. The values represent the median and range of the protein expression performed in triplicate assays using lymphocytes obtained from three different healthy donors or three patients with breast cancer.** a** Lymphocytes control, circulating T-lymphocytes collected from healthy woman (woman not affected by cancer;** b** Lymphocytes patient, circulating T-lymphocytes obtained from breast cancer patient; +HS, T-lymphocytes incubated with 50 μg/mL of heparan sulfate (HS), during 4 h at 37 °C. Kruskal–Wallis test with Dunn auxiliary test, *p < 0.0001

Heparan sulfate increased the expression of both heparanases in control circulating T-lymphocytes (Fig. 3a). However, such a stimulatory effect was not observed in breast cancer patients’ T-lymphocytes (Fig. 3b).

These combined results suggest that heparan sulfate/Syn-1 might be involved in T-lymphocyte activation, leading to increases in the expression of heparanase isoforms.

**Fig. 4:**
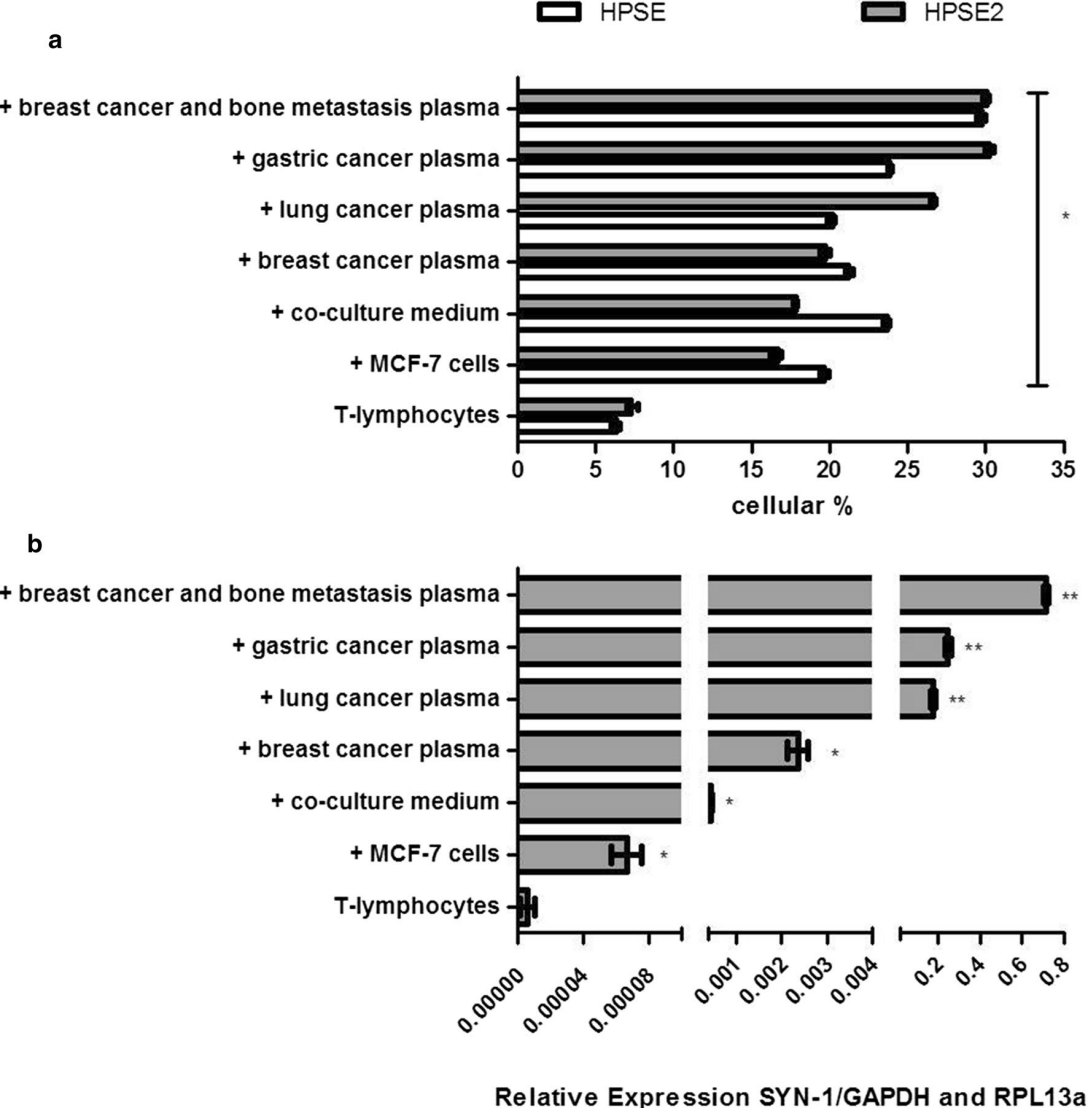
Expression of heparanases and syndecan-1. **a** Flow cytometer analysis to detect heparanase isoforms (HPSE and HPSE2) in circulating T-lymphocytes. T-Lymphocytes, lymphocytes collected from a healthy woman; T-lymphocytes were activated during 4 h at 37 °C as indicated in the presence of, MCF-7 cells (+ MCF-7 cells); conditioned medium collected from co-culture (+ co-culture medium); plasma obtained from breast cancer patient (+ breast cancer plasma); plasma obtained from lung cancer patient (+ lung cancer plasma); plasma from gastric cancer patient (+ gastric cancer plasma); plasma from breast cancer patient that has bone metastasis (+ breast cancer and bone metastasis plasma). All values represent the median and standard deviation performed in triplicate assays. The analyses were performed using Kruskal–Wallis test with Dunn auxiliary test. *HPSE/HPSE2 expression compared to the expression of non-activated T-lymphocytes, *p < 0.0001; **b** mRNA expression of heparan sulfate proteoglycan syndecan-1 (SYN-1) in circulating T-lymphocytes obtained by qPCR analysis. The assays were performed using lymphocytes collected from five healthy donors and plasma samples collected from four different patients with diverse types of cancer. Therefore, the values represent median and range of the triplicate experiments. RT-PCR reaction was performed in quadruplicates for each independent sample. */**syndecan-1 expression compared to the expression obtained of non-activated T-lymphocytes, *p < 0.005 and **p < 0.0001

Increased protein levels of both heparanase isoforms and an enhanced Syn-1 mRNA expression in circulating T-lymphocytes were observed after exposure to the plasma obtained from patients with different types of cancer, as shown in Fig. 4.

**Fig. 5:**
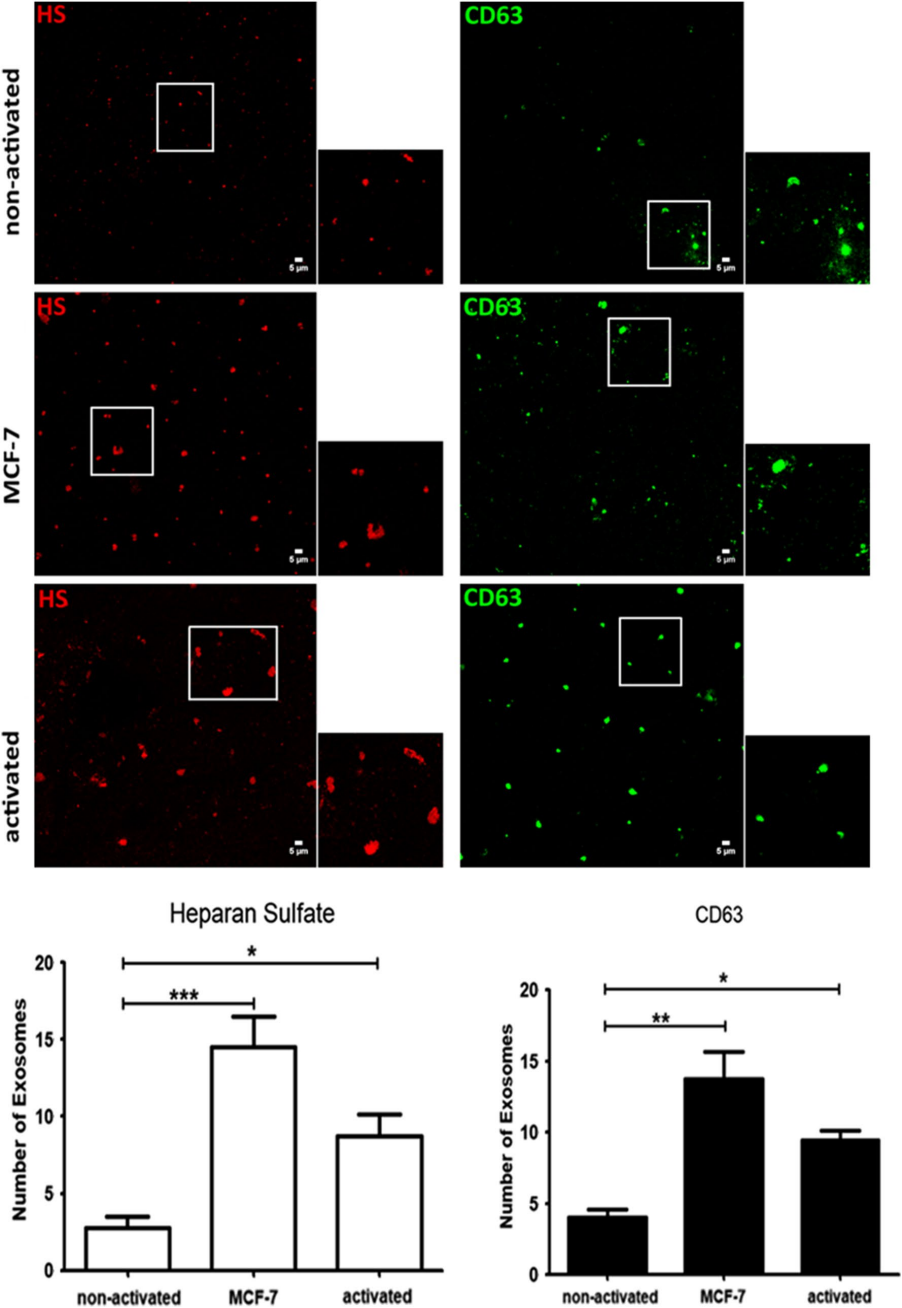
Exosome analysis. The exosomes were purified from collected medium of a healthy woman lymphocytes, co-culture lymphocytes (lymphocytes and MCF-7 cells) or MCF-7 cells, respectively, non-activated medium, MCF-7 medium and activated medium. The exosomes were immobilized in glass slides by cytocentrifugation and subsequently permeabilized with 0.01% saponin and analyzed by confocal microscopy after anti-heparan sulfate and anti-CD63 labeling. Heparan sulfate was revealed using a secondary antibody conjugated with Alexa Fluor^®^647 (red fluorescence), while exosomes were labelled using anti-CD63 conjugated with FITC (green fluorescence). The ROI images correspond to 1.45 × zoom. The values expressed in the graphs indicate the number of exosomes labelled with heparan sulfate antibody (red) and anti-CD63 (green). Bars in wide field images: 5 μm. ROI: region of interest. Statistical analysis was performed using One-Way analysis of variance with Dunnett´s multiple comparison test. */*** number of exosomes (Heparan sulfate labelling) comparing activated lymphocytes to the non-activated lymphocytes and MCF-7 medium to the non-activated lymphocytes, */***p < 0.05. */** number of exosomes (CD63 labelling) comparing activated lymphocytes to the non-activated lymphocytes and MCF-7 medium to the non-activated lymphocytes, */**p < 0.05. The experiment was performed using lymphocytes obtained from one healthy donor which is shared in two samples, lymphocytes non-activated and lymphocytes activated with MCF-7 cells (co-culture lymphocytes)

The evaluation of the exosome preparations by confocal microscopy demonstrated that breast cancer cells (MCF-7), as well as activated T-lymphocytes, presented an increased number of exosomes (based on CD63 labeling). Furthermore, the amount of heparan sulfate was higher in the exosomes obtained from MCF-7 cells and activated T-lymphocytes compared to those of non-activated T-lymphocytes (Fig. 5).

**Fig. 6:**
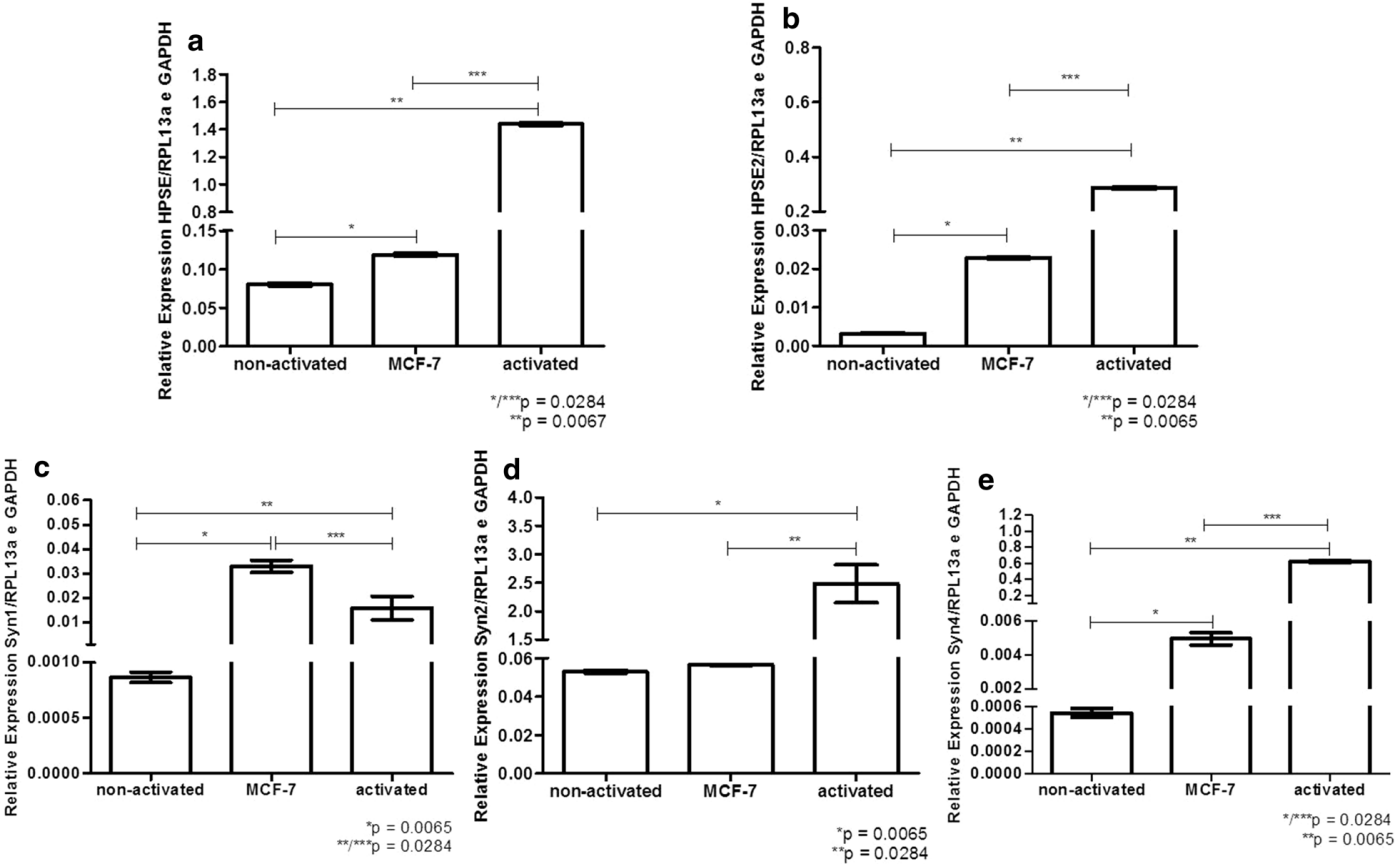
Relative expression of heparanases and syndecans in the exosomes. A pool of lymphocytes obtained from two healthy women donors was used to obtain non-activated lymphocytes or lymphocytes that were co-cultured with MCF-7 cells (activated lymphocytes). Exosomes were purified from conditioned medium of (non-activated T-lymphocytes); breast cancer cell line (MCF-7); or (activated T-lymphocytes). It is important to observe that T-lymphocytes activation was performed during 4 h at 37 °C and subsequently lymphocytes were plated in culture with DMEM medium, containing 10% fetal bovine serum and maintained overnight at 37 °C. The supernatant containing the cell-free media was used to obtain exosomes using Total Exosome Isolation kit. Quantitative RT-PCR was performed using total RNA extraction purified from exosomes preparation. **a** heparanase enzyme (HPSE); **b** heparanase-2 (HPSE2); **c** syndecan-1 (Syn1); **d** syndecan-2 (Syn2); **e** syndecan-4 (Syn4). The constitutive endogenous genes RPL13a and GAPDH were used to obtain the relative expression. The values represent average and standard deviation of triplicate assays. Statistical analysis was performed using Kruskal–Wallis test with Dunn auxiliary test

Figure 6 shows that exosomes secreted by MCF-7 and activated T-lymphocytes had increased expression of HPSE (Fig. 6a), HPSE2 (Fig. 6b), Syn-1 (Fig. 6c), and Syn-4 (Fig. 6e) mRNA compared to those of non-activated lymphocytes. Moreover, Syn-2 (Fig. 6d) mRNA was equally expressed in MCF-7 cells and non-activated lymphocytes, while it was overexpressed in activated lymphocytes.

It is important to mention that the same expression profile of heparanases and syndecans was found after treatment of exosomes with RNase, indicating that the expression of such molecules is due to RNA molecules contained inside of exosomes (data not shown). Additionally, the treatment of co-culture medium with anti-Syn-1 reduced the expression of both heparanases (Fig. 2), suggesting that Syn-1 may have been carried on the surface of the exosomes. It is also possible that other proteoglycans, and even heparanase, could be carried by exosomes.

**Fig. 7:**
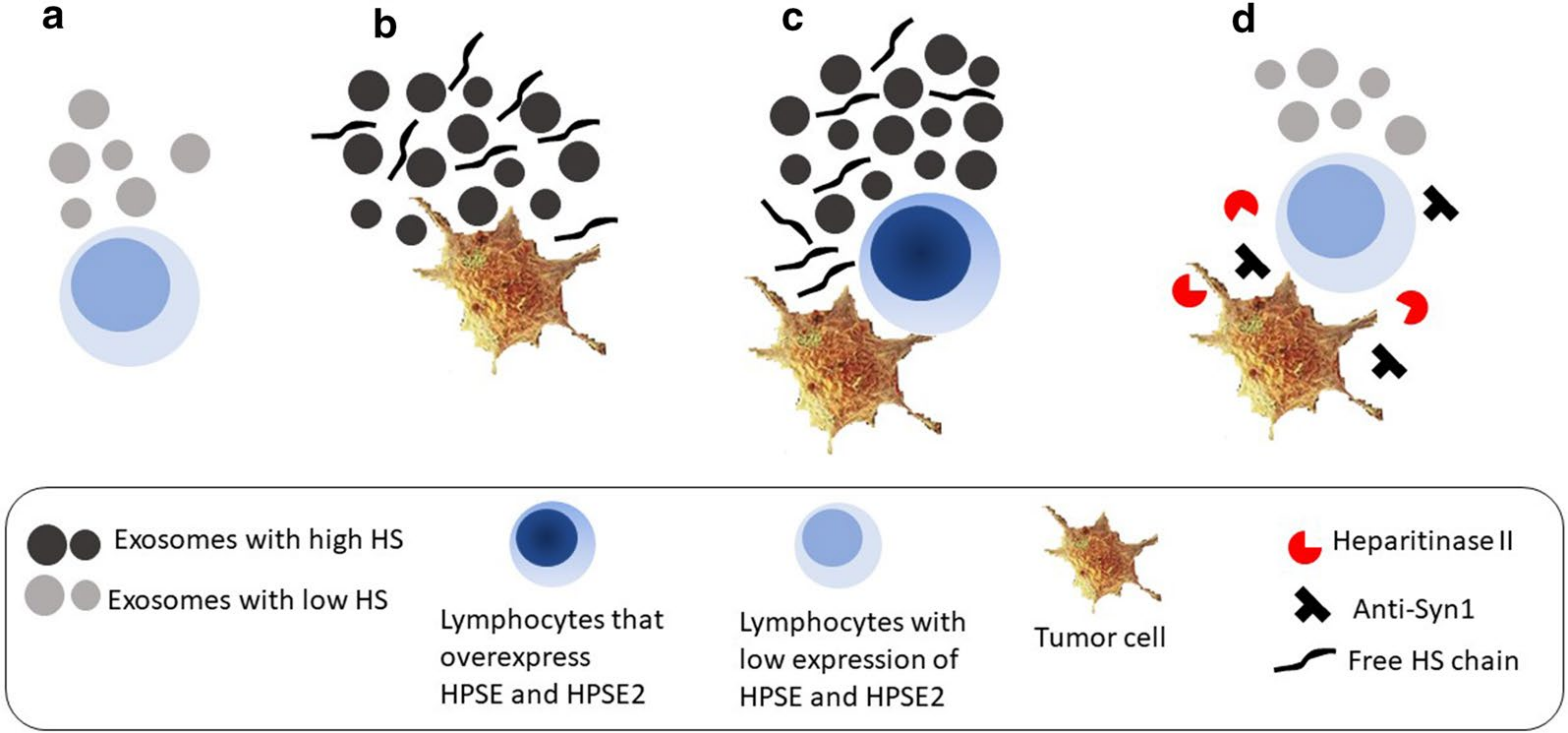
Induction of heparanase (HPSE) and heparanase-2 (HPSE2) expression in circulating lymphocytes by tumor cells. **a** circulating lymphocytes present low expression of HPSE and HPSE2 and secrete low number of exosomes with a low content of heparan sulfate (HS); **b** tumor cells secrete a larger number of exosomes which have higher content of HS, as well as higher number of HS free chains; **c** lymphocytes in the presence of tumor cells (co-culture) express higher level of HPSE and HPSE2 and also secrete an increased number of exosomes and free heparan sulfate chains compared to lymphocytes that do not contact tumor cells; **d** lymphocytes incubated with co-culture conditioned medium in the presence of anti-heparan sulfate proteoglycan, syndecan-1 (anti-syn1), as well as in the presence of the enzyme heparitinase II express exosomes with low level of HPSE and HPSE2 and less amount of HS

The scheme depicted in Fig. 7 indicates that tumor cells secreted a greater number of exosomes with high HS content and free HS chains, which generated a microenvironment capable of activating the expression of HPSE and HPSE2 in circulating lymphocytes. Furthermore, it was also observed that exosomes secreted by circulating lymphocytes that were in contact with the tumor microenvironment expressed a greater amount of mRNA of both heparanases and of heparan sulfate proteoglycans, as well as more heparan sulfate compared to that of lymphocytes that did not have contact with tumor cells. Additionally, activated lymphocytes and tumor cells secreted free chains of heparan sulfate, while lymphocytes obtained from healthy donors did not secrete free heparan sulfate. It is well known that the overexpression of the HPSE enzyme is directly related to carcinogenesis. The present results indicate that tumor cells have a crosstalk with circulating lymphocytes to positively modulate the expression of HPSE. The heparan sulfate secreted by activated lymphocytes and tumor cells, as well as heparan sulfate present in circulating exosomes, may promote the development of distantly located tumors by inducing heparanase expression.

## Discussion

Breast cancer cells (MCF-7) and co-culture medium were able to upregulate both heparanase isoforms in circulating lymphocytes, mimicking the effect of plasma from breast cancer patients.

The glycosaminoglycan profile clearly demonstrated that the circulating lymphocytes from breast cancer patients secreted heparan sulfate. Conversely, the lymphocytes from healthy women did not secrete this molecule. Syndecan-1 is present in malignant cells. Elevated levels of Syn-1 in different tumors and its increased shedding correlated with an unfavorable prognosis for patients with malignant neoplasms [21]. The presence of secreted heparan sulfate in the culture medium of breast cancer patient samples and the absence of this glycosaminoglycan in the culture medium of samples from healthy women reinforced the idea that heparan sulfate secreted by circulating lymphocytes can modulate the molecular mechanisms of carcinogenesis.

The fact that the anti-Syn-1 antibody blocked the stimulatory effect of both heparanases in lymphocytes suggested that this proteoglycan might modulate the expression of the different heparanase isoforms. Moreover, the degradation of heparan sulfate chains abolished the stimulatory effect that was observed on heparanase expression in circulating lymphocytes, indicating that this effect was dependent on the presence of heparan sulfate chains.

It was previously shown that more aggressive tumors exhibit higher heparanase and Syn-1 expression compared to that of nonmetastatic tumors, suggesting that targeting heparanase might improve cancer treatment [31, 36]. Syndecan-1 is associated with breast cancer cell adhesion, migration, and resistance [37, 38]. Furthermore, Syndecan-2 and Syndecan-4 seem to be important regulators of breast carcinoma progression [39]. The increased levels of Syn-1 observed in breast cancer lymphocytes, after lymphocyte exposure to the plasma of breast cancer patients and after co-culture with MCF-7 cells suggested that Syn-1 shedding could induce heparanase expression. The present results showed a direct correlation between the presence of Syn-1 and heparan sulfate and the upregulation of heparanase isoforms.

The heparan sulfate chains from Syn-1 might induce HPSE and HPSE2 expression in circulating lymphocytes as well as exogenous heparan sulfate-free chains.

The plasma obtained from patients with different types of cancer was able to elicit an upregulation of HPSE, HPSE2 and Syn-1 mRNA expression. The higher levels of circulating heparan sulfate found in the plasma of patients with cancer could explain such stimulatory effect.

The heparan sulfate-activated lymphocytes overexpressed HPSE, and this might have increased cellular migration, which is highly relevant for tumor metastasis.

Very little is known regarding the regulation of exosome production and secretion by tumor cells. It has been demonstrated that the heparanase enzyme dramatically upregulates exosome secretion [27–29]. It was also observed that recombinant heparanase stimulated exosome secretion from breast carcinoma cells. The abundant exosomes are recognized by receptors that facilitate heparanase internalization; once cells take up heparanase, the enzyme triggers specific biological functions involved in tumor development [27].

Taken together, the results obtained in the present study suggest that the heparanase enzyme (HPSE) released by tumor cells could diffuse within the microenvironment and impact neighboring tissues, such as circulating lymphocytes. Moreover, increased HPSE expression in circulating lymphocytes and tumor cells possibly stimulates exosome secretion, thereby promoting tumor progression.

It has been shown that the heparan sulfate that was present in exosomes was able to exert a stimulatory effect on heparanase expression [26]. Our results corroborated the literature and provided evidence of an increased number of exosomes as well as increased levels of heparan sulfate inside exosomes from tumor cells (MCF-7) or from lymphocytes activated by tumor cells compared to those of the non-activated lymphocytes.

We propose that the increased expression of heparanase isoforms in circulating lymphocytes might be modulated by Syn-1. It is possible that the mechanism of syndecan-1/heparan sulfate modulation in the tumor cell microenvironment could be mediated by exosomes.

The overexpression of HPSE2 in circulating lymphocytes of breast cancer patients has previously been described by our group [18]. The present study provides unprecedented evidence that HPSE2 expression can also be modulated by the crosstalk with tumor cells. Therefore, mechanisms that modulate HPSE expression appear to be similar to those that modulate HPSE2 expression. Moreover, the role of HPSE2 in carcinogenesis is still the subject of investigation, and it has been suggested that HPSE2 is involved in the inhibition of HPSE activity, regulates selected genes that promote normal differentiation, endoplasmic reticulum stress, tumor fibrosis, apoptosis and is a tumor suppressor [40].

## Conclusions

The present study elucidated the molecular mechanisms involved in the activation of heparanase expression in circulating lymphocytes, which was mediated by heparan sulfate and syndecans that were secreted in exosomes by tumor cells. The interaction of lymphocytes with tumor cells is essential for the stimulation of the secretion and for changes in the mRNA profile of exosomes. The combined results explained the influence of the tumor microenvironment in carcinogenesis, which led to insight into the possible molecules, such as syndecan-1 and heparanase, which could be used as targets for tumor prognosis and as future supplementary treatment for cancer.

## Abbreviations

BL: triple negative molecular subtype basal-like
BMDC: bone marrow-derived cell
DMEM: Dulbecco’s Modified Eagle Medium
ECM: extracellular matrix
FBS: fetal bovine serum
HPSE: heparanase-1
HPSE2: heparanase-2
HS: heparan sulfate
HSPG: heparan sulfate proteoglycan
IM: triple negative molecular subtype immunomodulatory
LAR: triple negative molecular subtype luminal androgen receptor
M: triple negative molecular subtype mesenchymal
MCF-7: established human breast cancer cell line luminal A
MDSC: myeloid cell-derived suppressor cell
miRNA: micro RNA
mRNA: messenger RNA
MSC: mesenchymal stem cell
NK cells: natural killer cells
PBMC: peripheral blood mononuclear cells
PDA: acetate buffer and 1,3-diaminopropane
PG545: synthetic mixture of oligosaccharides derived from heparin)
PI88: high sulfonated mannan oligosaccharide
PMA: phorbol ester
RT-PCR: reverse transcription followed by polymerase chain reaction
SST0001 or Roneparstat: modified heparin saccharide N-acetylated
Syn-1: syndecan-1
